# Prevalence of the *TP53* p.R337H Mutation in Breast Cancer Patients in Brazil

**DOI:** 10.1371/journal.pone.0099893

**Published:** 2014-06-17

**Authors:** Juliana Giacomazzi, Marcia S. Graudenz, Cynthia A. B. T. Osorio, Patricia Koehler-Santos, Edenir I. Palmero, Marcelo Zagonel-Oliveira, Rodrigo A. D. Michelli, Cristovam Scapulatempo Neto, Gabriela C. Fernandes, Maria Isabel W. S. Achatz, Ghyslaine Martel-Planche, Fernando A. Soares, Maira Caleffi, José Roberto Goldim, Pierre Hainaut, Suzi A. Camey, Patricia Ashton-Prolla

**Affiliations:** 1 Genomic Medicine Laboratory, Experimental Research Centre, Hospital de Clínicas de Porto Alegre (HCPA), Porto Alegre, Rio Grande do Sul, Brazil; 2 Post-Graduate Program in Medicine: Medical Sciences, Universidade Federal do Rio Grande do Sul (UFRGS), Porto Alegre, Rio Grande do Sul, Brazil; 3 Pathology Service, HCPA, Porto Alegre, Rio Grande do Sul, Brazil and Instituto de Patologia, Porto Alegre, Rio Grande do Sul, Brazil; 4 Pathology Service, Hospital do Câncer AC Camargo (HCACC), São Paulo, São Paulo, Brazil; 5 Protein and Molecular Analysis Laboratory, Experimental Research Centre, HCPA, Porto Alegre, Rio Grande do Sul, Brazil; 6 Molecular Oncology Research Centre, Hospital do Câncer de Barretos, Barretos, São Paulo, Brazil; 7 National Institute of Populational Medical Genetics (INAGEMP), UFRGS, Porto Alegre, Rio Grande do Sul, Brazil; 8 Oncogenetics Department, HCACC, São Paulo, São Paulo, Brazil; 9 International Agency for Research on Cancer (IARC), Lyon, Rhone, France; 10 Hospital Moinhos de Vento, Porto Alegre, Rio Grande do Sul, Brazil; 11 Bioethics Research Laboratory, HCPA, Porto Alegre, Rio Grande do Sul, Brazil; 12 International Prevention Research Institute, Lyon, Rhone, France; 13 Department of Statistics, Institute of Mathematics, UFRGS, Porto Alegre, Rio Grande do Sul, Brazil; 14 Post-Graduate Program in Genetics and Molecular Biology, UFRGS, Porto Alegre, Rio Grande do Sul, Brazil; Cancer Research Centre of Lyon, France

## Abstract

Germline *TP53* mutations predispose individuals to multiple cancers and are associated with Li-Fraumeni/Li-Fraumeni-Like Syndromes (LFS/LFL). The founder mutation *TP53* p.R337H is detected in 0.3% of the general population in southern Brazil. This mutation is associated with an increased risk of childhood adrenal cortical carcinoma (ACC) but is also common in Brazilian LFS/LFL families. Breast Cancer (BC) is one of the most common cancers diagnosed in *TP53* mutation carriers. We have assessed the prevalence of p.R337H in two groups: (1) 59 BC affected women with a familial history (FH) suggestive of hereditary cancer syndrome but no LFS/LFL features; (2) 815 BC affected women unselected for cancer FH, diagnosed with BC at or before age 45 or at age 55 or older. Among group 1 and group 2 patients, 2/59 (3.4%, CI95%: 0.4%–11.7%) and 70/815 (8.6%, CI95%: 6.8%–10.7%), respectively, were p.R337H carriers in the germline. The prevalence of p.R337H was higher in women diagnosed with BC at or before age 45 (12.1%, CI95%: 9.1%–15.8%) than at age 55 or older (5.1%, CI95%: 3.2%–7.7%), p<0.001). The Brazilian founder p.R337H haplotype was detected in all carriers analysed. These results suggest that inheritance of p.R337H may significantly contribute to the high incidence of BC in Brazil, in addition to its recently demonstrated impact on the risk of childhood ACC.

## Introduction

Germline *TP53* mutations are the underlying genetic defect in Li-Fraumeni Syndrome (LFS) and its variant, Li-Fraumeni-Like Syndrome (LFL), autosomal dominant disorders characterised by a predisposition to multiple early-onset cancers [1]. The most frequent tumours in LFS/LFL are adrenal cortical carcinoma (ACC), soft tissue and bone sarcomas, brain tumours, and breast cancer (BC). BC is the most common cancer in adult *TP53* mutation carriers, representing over 25% of all cancer diagnoses [1], [2].

In Europe and North America, germline *TP53* mutations have been estimated to occur in 1 of 5,000–20,000 live births. Among BC-affected women, mutations have been described in no more than 0.25% of those diagnosed with BC at any age and unselected for familial history of cancer (FHC) [2], [3], and in up to 7% of those with very early-onset BC (diagnosis before age 30), independent of FHC [1], [4]–[8]. A recent study in a cohort of 100 BC patients diagnosed at or before age 35 identified germline *BRCA1*, *BRCA2* and *TP53* mutations in 11, 6, and 5% of the patients, respectively, supporting that the *TP53* mutation screening should be offered together with *BRCA1/2* testing in women with early-onset BC [7].

In southern and southeastern regions of Brazil, a specific mutation that occurs in codon 337 (g.16901G>A; p.R337H), has been reported to occur at a high frequency [9]–[14]. The arginine residue at codon 337 is part of an alpha-helix motif involved in p53 oligomerisation and structural studies have shown that replacement of arginine by histidine disrupts oligomerisation in a pH-dependent manner, making the domain unable to oligomerise in conditions of slightly elevated pH [15]. Although biological dependence upon pH has not been demonstrated *in vivo* thus far, it is plausible that the p.R337H mutant protein operates as a conditional mutant. Of note, while normal cells maintain an intracellular pH gradient that is slightly more acidic than extracellular pH, many cancer cells show a reversed pH gradient with a constitutively increased, slightly alkaline intracellular pH [16]. This reverse pH gradient contributes to activate glycolytic enzymes such as LDH (lactate dehydrogenase). Since wild-type p53 activity represses several components of glycolysis it is plausible that the dependency of p.R337H activity upon pH may facilitate the metabolic adaptation of cancer cells to aerobic glycolysis, a well-defined cancer hallmark [17].

Independent studies have estimated the prevalence of p.R337H to be 0.28–0.30% in the general population of Southern Brazil [9], [12]. Haplotype studies have shown that the mutation occurs on the same *TP53* haplotype in all Brazilian p.R337H carriers, providing evidence of a founder effect [18]. A mass newborn screening program conducted in the State of Paraná (2005–2010) had tested 171,641 newborns, unselected for FHC, and 461 (0.27%) were found to be carriers of p.R337H and 11 of them (2.4%) had developed ACC. Evaluation of FHC in mutation carriers reported that 27.4% had FHC matching LFL criteria and 41.6% had FHC that fitted no clinical criteria for a cancer syndrome. Aside from this mass screening initiative, patients carrying the p.R337H mutation have been identified in families recruited in high risk cancer clinics and matching clinical definitions of LFS/LFL with ACC, soft tissue sarcoma, osteosarcoma, brain tumors and BC [13]. The p.R337H mutation has also been reported in individual cases with choroid plexus tumors (CPT), osteosarcoma and BC [9]–[12], [14].

Compared with mutations that alter the DNA binding domain of p53, which represent over 90% of missense *TP53* mutations in LFS/LFL families outside Brazil, the p.R337H mutation appears to be less penetrant [18]. BC represents 28.6% of all cancers in families with germline p.R337H mutation [11], compared to 27.2% and 27.8% in families with germline *TP53* mutations from Northern America and in Western Europe, respectively [19]. Interestingly, in two small-size case-control studies of BC-affected women recruited from southeastern Brazil, p.R337H was detected in 2.4% (3/123) and 0.5% (2/390) in BC cases but was not detected in the controls [20], [21]. In the present study, we assessed the prevalence of germline p.R337H in women diagnosed with BC at different ages, with or without a documented FHC and recruited from different regions of Brazil.

## Methods

### Recruitment and Patients

The manuscript describes 2 different approaches and 2 independent study groups. Group 1 included unrelated breast cancer patients that were recruited prospectively after written informed consent in a public University Hospital in Southern Brazil (Hospital de Clinicas de Porto Alegre). These patients had either a FH of hereditary breast and ovarian cancer (HBOC) and/or of breast and colon cancer (HBCC) syndromes and had no LFS/LFL criteria at recruitment [22], [23]. The decision of studying *TP53* p.R337H prevalence in families with the HBOC and HBCC phenotypes was influenced by recent reports describing germline mutations in *TP53* among families with breast and/or ovarian and breast and/or colorectal cancer [24], [25]. At recruitment, none of the patients fulfilling HBOC or HBCC criteria had been conclusively tested for *BRCA1/2* or *CHEK2* germline mutations. Group 2 included a retrospective collection of tissues. Consecutive series of breast cancer patients diagnosed in the pathology departments of 3 different academic centers in Brazil during a given period of time were identified. The sole purpose of the analysis was to determine mutation prevalence. This procedure was approved by the 3 institutional IRBs from the institutions were the samples were obtained before study initiation (Hospital de Clinicas de Porto Alegre and Instituto de Patologia – recruitment center (RC) 1, Hospital do Cancer AC Camargo – RC 2 and Hospital de Cancer de Barretos – RC 3 (Figure S1 in File S1). The samples were anonymized in each centre and all identifiers were completely removed before any genotyping analysis. Samples were sent to one laboratory (Hospital de Clinicas de Porto Alegre) for analysis. In addition to a confirmed histopathological diagnosis of BC, their age at diagnosis was the sole inclusion criterion for this group. Patients in group 2 were classified into two sub-groups: those diagnosed at or before age 45 (the upper age limit for BC according to the Revised Chompret Criteria for LFL) and those diagnosed at age 55 or older (post-menopausal BC). Breast cancer patients diagnosed between the ages of 45–55 years were not included in order to minimize inclusion of patients with a less well-defined menopausal status.

### Tissue Samples

Haematoxylin-eosin (H&E) slides of all BC cases in Group 2 were re-assessed by three pathologists (MSG, CABTO, CSN) to confirm the tumour type and grade. Formalin-fixed paraffin-embedded (FFPE) non-tumoural lymph node tissue, fresh frozen tumoural tissue (whenever available) and histopathological reports containing information on tumour type, grade and immunohistochemistry were obtained and were relabelled to remove individual identifiers before sample excision (1·0-mm punch directly) and genotyping.

### Genotyping for p.R337H

DNA samples of group 1 patients were obtained from peripheral blood using a commercial DNA extraction kit (Illustra Blood genomicPrep Mini Spin Kit, GE Healthcare) and of group 2 patients from non-tumoural and tumoural tissues using a DNA FFPE Kit (Qiagen). Genotyping for p.R337H was performed by allelic discrimination using qPCR-TaqMan (Applied Biosystems). All mutation-positive samples and a randomly selected sub-sample of 150 (20%) mutation-negative samples were confirmed by a second, independent qPCR analysis. The accuracy of genotyping by qPCR was confirmed by the direct Sanger sequencing of *TP53* exon 10 in a subset of 65 samples (35 mutation-positive and 30 mutation-negative samples) by qPCR, using the primers and protocols previously described [19]. Homozygous mutants were confirmed by PCR-RFLP (Figure S2 in File S1) [12]. Genotyping of the tumour DNA was performed to assess loss of heterozygosity (LOH) at the p.R337H locus whenever possible. The presence of the Brazilian founder p.R337H haplotype was confirmed in the mutation-positive samples by ASO-PCR and nested-PCR analysing SNP 15 (rs1642785) and SNP28 (rs9894946) from a panel of 29 intragenic SNPs, as described elsewhere [18].

### Geo-Mapping

All Group 1 patients were from the State of Rio Grande do Sul, Brazil. Group 2 patients were from different Brazilian regions and were geo-coded using informed residence zip codes associated with a base map provided by the Brazilian Institute of Geography and Statistics (IBGE) [26]. Corresponding statistical certainty geo-maps were produced using the software ArcGIS 10.0 ESRI.

### Statistical Analyses

SPSS version 16.0 was used for the data handling and the statistical analyses. The categorical variables were described by their absolute and/or relative frequencies, quantitative variables were expressed as the mean ± the standard deviation (SD) or the median and the minimum-maximum, and t-test was used to compare mean values for independent groups. Associations were tested by Fisher’s exact test. In all analyses, a significance level of 0.05 was adopted.

## Results

**Figure 1:**
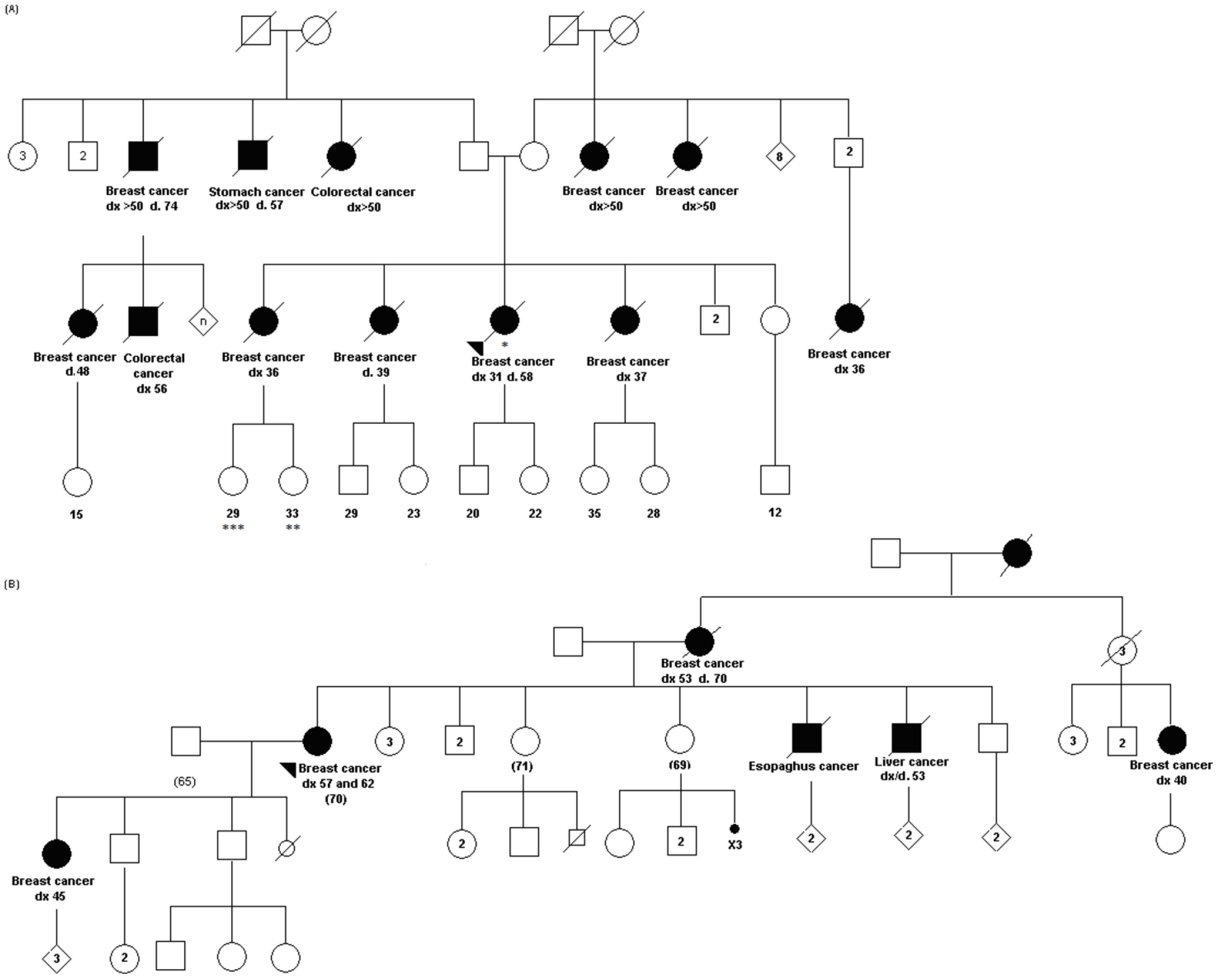
Pedigrees of mutation-positive probands from group 1. Blackened symbols represent cancer-affected relatives. An arrow indicates the proband. Dx: age at diagnosis; WT: wild-type; WT: wild-type; *TP53 (p.R337H) mutation carriers and negative for *BRCA1/2*; **TP53 (p.R337H) and *BRCA1* negative and *BRCA2* mutation carrier; ***TP53 (p.R337H) negative an *BRCA1/2* negative.

The mean age at BC diagnosis for patients in Group 1 was 43 years (SD = 11.9). Of the 59 patients recruited for this study group, 54 met HBOC criteria and 5 met both HBOC and HBCC criteria. Two patients (3.4%) were p.R337H carriers. The first met HBOC and HBCC criteria and was diagnosed with breast cancer at age 31 years. The second met only HBOC criteria and was diagnosed with BC at age 57 years. Interestingly, in this latter family a child was subsequently diagnosed with ACC and the family history now fulfils also Modified Chompret criteria. Both probands were tested for germline *BRCA1* and *BRCA2* mutations and found to be negative. Interestingly, however, a pathogenic *BRCA2* mutation was found in a second-degree relative of the first proband (Figure 1). Additional detailed data on all molecular testing results available (such as *BRCA1, BRCA2* and *CHEK2* mutation status of the familial cases) are summarized in Table S1 in File S1.

Group 2 included 403 patients diagnosed with BC at or before age 45 and 412 patients diagnosed at age 55 or older. The mean ages at diagnosis in these two groups were 38 (SD = 5) and 66 (SD = 9) years, respectively. The majority of patients had invasive carcinomas (n = 738; 90.5%). Overall, genotyping identified p.R337H in 8.6% of all individuals genotyped. Mutation frequency was inversely associated with age; 12.1% of the women in the group of cases diagnosed at or before age 45 years were p.R337H-positive, and 5.1% of women diagnosed at age 55 or older were found to be mutation carriers (p<0.001) (Table 1). For women diagnosed at or before age 30, the carrier rate was 20% (8/40, CI95%: 9.0%–35.6%). Of the mutation-positive cases, 68 (97.1%) were heterozygote carriers (c.1010 AG). In two cases, only mutant alleles were detected, suggesting that these patients were constitutive mutant homozygotes or hemizygotes (CNV with LOH at the *TP53* p.R337H locus cannot be excluded). In 23 of the *TP53* p.R337H carriers available biological materials enabled mutation screening in all coding exons (2–11) of the gene and no other deleterious mutations were identified. The Brazilian founder haplotype was detected in all of the p.R337H carriers who were analysed (n = 22).

**Table 1:** *TP53* p.R337H mutation status reported according to the patient’s age at BC diagnosis and the recruitment centre (n = 815).

	RC 1 (n = 293)	RC 2 (n = 238)	RC 3 (n = 284)	Total (n = 815)
**p.R337H carriers**				
**Age at BC diagnosis**	**“n” carriers/n total (%)**			
≤45 yrs	14/136 (10·3%)	33/123 (26·8%)	2/144 (1·4%)	49/403 (12·1%)
≥55 yrs	3/157 (1·9%)	17/115 (14·8%)	1/140 (0·7%)	21/412 (5·1%)
**p.R337H non-carriers**				
**Age at BC diagnosis**	**“n” non-carriers/n total (%)**			
≤45 yrs	122/136 (89·7%)	90/123 (73·2%)	142/144 (98·6%)	354/403 (87·4%)
≥55 yrs	154/157 (98·1%)	98/115 (85·2%)	139/140 (99·3%)	391/412 (94·9%)

Legend: n-number of subjects; RC-recruitment centre: 1-Porto Alegre, 2-São Paulo and 3-Barretos; BC-breast cancer.

**Figure 2:**
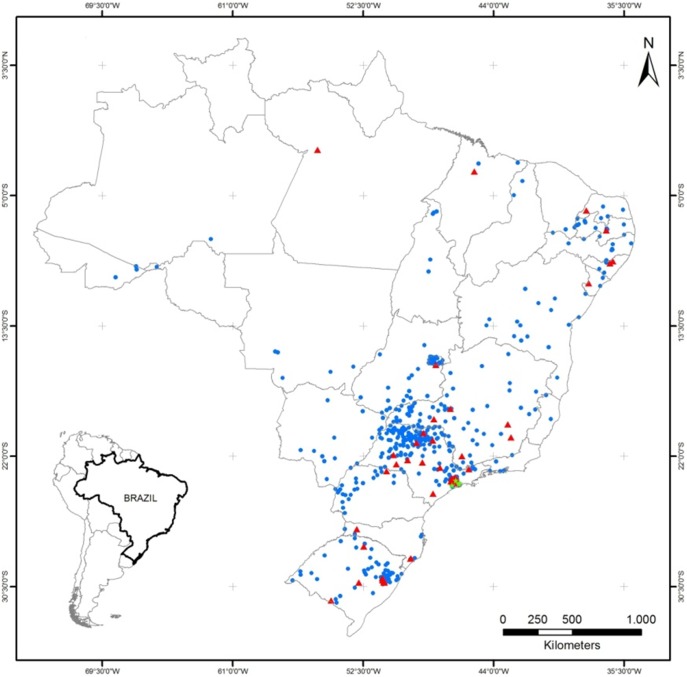
The geographic distribution of breast cancer-affected women in group 2 whose city of residence was known (n = 657). Legend: Blue dots, red triangles and green stars represent the cities of residence of women who are homozygous normal, heterozygous, and homozygous mutant p.R337H, respectively.

Geographical distribution of 657 (80.5%) BC-affected women of group 2, colour-coded according to mutation status and RC, were plotted onto a map of Brazil using the zip code for each subject’s place of residence at the time of diagnosis. The mutation carriers were widely distributed among the catchment areas of the three RC (Figure 2). Overall, p.R337H was detected in 13/216 (6.0%, CI95%: 3.2%–10.1%), 35/291 (12.0%, CI95%: 8.5%–16.3%) and 5/97 (5.1%, CI95%: 1.7%–11.6%) women residing in the southern, southeastern and central/northern regions of Brazil, respectively (p = 0.04). Haplotyping of three of the five patients residing in the northern regions confirmed the presence of the Brazilian founder allele.

## Discussion

The mutant p.R337H founder allele has been identified in approximately 0.3% of the general population of southern Brazil in two independent prevalence studies carried out with equivalent genotyping strategies in densely populated areas located over 1,000 km apart [12], [13], which are within the same geographical regions from where a proportion of individuals of the present study derive. Mass newborn screening conducted in the State of Paraná, Southern Brazil, had identified 461 carriers among 171.641 newborns (0.27%), 11 of whom (2.4%) had subsequently developed ACC, the earliest sentinel cancer of LFS/LFL. Given that BC represents the most common cancer in female *TP53* mutation carriers, this study has been designed to determine whether, similar as ACC, p.R337H may be a risk factor for BC, the main sentinel cancer for adult LFS/LFL. Studies in families reporting to high-risk cancer clinics have shown that BC represents 28.6% of all cancers diagnosed in p.R337H carriers [11]. However, given the partial penetrance and the wide variations in the patterns of inherited cancer in carriers of this mutation, it is plausible that a significant proportion of BC in Brazilian women may occur in a background of germline p.R337H carriers. In this study, we have identified the germline *TP53* mutation in a high percentage (8.6%) of BC-affected women unselected for a FHC. In this group (group 2) the mutation was more common in women with pre-menopausal than post-menopausal BC and mutation frequency reached 20% in those diagnosed with BC at or before age 30. Furthermore, it appears to be more frequent in patients residing in the southern and south-eastern regions of the country.

On the other hand, even in a series of women with nosological definitions of BC syndromes other than LFS/LFL (group 1), p.R337H was detected at a significant frequency for a single mutation (of 3.4%). These findings identify p.R337H as the most common germline *TP53* mutation associated with cancer described in any population and as the single most prevalent cancer-associated founder alleles identified to date.

The association between p.R337H and early, pre-menopausal BC is consistent with the tumour patterns observed in subjects who carry other germline mutant *TP53* alleles. In families who meet clinical criteria for LFS, the mean age at BC diagnosis is 32·35 years [27].

It is interesting to note that the association between p.R337H and BC is not restricted to pre-menopausal BC. It occurs at a lower prevalence, in women diagnosed with BC at age 55 or older. Several studies have investigated the prevalence of germline *TP53* mutations in non-Brazilian BC patients selected or not selected for a FHC, providing evidence that germline *TP53* mutations are associated with a fraction of early BC diagnoses irrespective of a FHC [4], [5], [28], [29]. However, it should be noted that in all these studies, the mutations identified are different and primarily, if not exclusively, occur in the DNA-binding domain of the p53 protein. Therefore, our study is unique in demonstrating the prevalence of a single mutation in Brazilian patients with early-onset BC that is greater than the reported prevalence of all other *TP53* mutations worldwide. Because patients in group 2 were unselected for a FHC, we can not, at present, determine the prevalence of the mutation in BC patients with and without a FHC. However, early-onset BC, regardless of FH, could be considered a sentinel phenotype for the identification of subjects who are carriers of germline p.R337H in Brazilian patients. Whereas some carriers may develop cancer in the absence of any suspicious FHC, it has to be expected that a significant proportion of these patients may have relatives at high risk for cancer. Indeed, in the population of 461 newborns who were identified as carriers in a mass screening intervention in the State of Paraná, 27.4% had a FH that matched LFL criteria whereas another 41.8% had a FH that did not match any known syndrome, compatible with the wide disparities of patterns of inheritance in p.R337H carriers.

Previous studies on Brazilian BC cohorts have analysed the prevalence of the p.R337H mutation [9]–[14], [20], [21]. However, compared to the present study, these studies were smaller in size and did not always provide details on the FHC or the geographic localisation of the patients. The results from the present study indicate that a significant geographic variation in mutation frequency may exist. Different reasons can explain this observation. First, there may be differences in the dissemination of the founder haplotype in certain regions of the country. Second, we can not exclude an ascertainment bias due to the wide socioeconomic disparities and the variable distribution of BC risk factors that may influence BC risk across Brazil. Finally, referral patterns in the different RCs may also have influenced our results. Referrals to RC1 are based on pathology laboratory in one of the highest-resources areas of Brazil, and may have an over-representation of BC with “western-type” risk factor profile. RC2 is based in a specialised cancer hospital, hosting the largest oncogenetics practice in the country recruiting a large proportion of patients with a FHC and may have an overrepresentation of cases with aggressive tumours that may not be easily managed in other centres. RC3 is also based in a specialised cancer hospital with an itinerant early detection and intervention program and may thus recruit a higher number of patients with small, early-stage cancers [30].

Another important question involves the mechanisms of breast carcinogenesis related to the p.R337H mutation because tumour genotyping revealed that loss of the wild-type allele (LOH) at the mutation locus is not common in these cases. Indeed this feature had been reported previously in a smaller study [18]. In contrast, in virtually all ACC and CPT of paediatric p.R337H carriers, LOH has been described at the mutation locus with the loss of the wild-type allele, consistent with the classic two-hit model proposed for carcinogenesis associated with tumour suppressor genes [9], [10], [12]. Thus, the mechanism of p.R337H-associated carcinogenesis may depend on the tissue and the patients age when the tumour diagnosis was made. It should be kept in mind, however, that p.R337H is a oligomerization domain mutant, and not a DNA-binding domain mutation. Therefore, once tuned to mutant phenotype in a pH-dependent manner, this protein may interfere with p53 oligomerization in a way that may prevent the assembly of wild-type p53 complexes, thus making it unnecessary to eliminate the wild-type allele through LOH. This hypothesis may also account for the observation that tumor patterns in subjects who have inherited two mutant alleles are not more severe than in heterozygote carriers [31]. While the exact disease-causing mechanism of the p.R337H mutation remains elusive, alternative hypotheses should be considered, such as the influence of environmental changes onto pH and occurrence of constant, more subtle changes to oligomerisation *in vivo* without the requirement for drastic pH changes.

In conclusion, the germline p.R337H may contribute to a significant proportion of the health burden associated with BC in Brazil, and its identification may have important implications for disease management and cancer risk counselling not only in childhood, due to the risk for ACC, but also in adults, due to its association with BC and possibly other adult onset tumors.

## Supporting Information

File S1 Figure S1, The geographic distribution of breast cancer-affected women in group 2 whose city of residence was known (n = 657), color-coded by the recruiting centre. Legend: Blue, green and orange dots represent women from recruiting centres 1, 2 and 3, respectively. Figure S2, The detection of p.R337H by PCR-RFLP (*Restriction length fragment polymorphism*) and sequencing in homozygous and heterozygous p.R337H BC-affected women. Legend: The DNA was amplified by PCR to generate a 238-base pair product encompassing exon 10 and the flanking splice sites. (a) RFLP analysis with *HhaI* (MPM = molecular weight marker; B = blank, the arrow indicates the uncleaved 238-base pair PCR product corresponding to the A allele). (b) Sequencing of a homozygous p.R337H carrier and (c) sequencing of a heterozygous p.R337H carrier (the underlined base corresponds to nucleotide 16,901 of the *TP53* gene). Table S1, *BRCA1, BRCA2 and CHEK2 1100delC* mutation status of the patients analyzed for these genes in the group 1. Legend: NT: not tested; *TP53 p.R337H mutation carriers. Molecular results of 30 cases of this group are not available.
(DOC)
