# Supplementary material for: Prevalence of the TP53 p.R337H Mutation in Breast Cancer Patients in Brazil
Source: PLoS One. 2014 Jun 17;9(6):e99893. doi: 10.1371/journal.pone.0099893 (PMC4061038; doi:10.1371/journal.pone.0099893)
Supplement: File S1 — Figure S1, The geographic distribution of breast cancer-affected women in group 2 whose city of residence was known (n = 657), color-coded by the recruiting centre. Legend: Blue, green and orange dots represent women from recruiting centres 1, 2 and 3, respectively. Figure S2, The detection of p.R337H by PCR-RFLP (Restriction length fragment polymorphism) and sequencing in homozygous and heterozygous p.R337H BC-affected women. Legend: The DNA was amplified by PCR to generate a 238-base pair product encompassing exon 10 and the flanking splice sites. (a) RFLP analysis with HhaI (MPM = molecular weight marker; B = blank, the arrow indicates the uncleaved 238-base pair PCR product corresponding to the A allele). (b) Sequencing of a homozygous p.R337H carrier and (c) sequencing of a heterozygous p.R337H carrier (the underlined base corresponds to nucleotide 16,901 of the TP53 gene). Table S1, BRCA1, BRCA2 and CHEK2 1100delC mutation status of the patients analyzed for these genes in the group 1. Legend: NT: not tested; *TP53 p.R337H mutation carriers. Molecular results of 30 cases of this group are not available. (DOC) [file pone.0099893.s001.doc]

**Figure S1. The geographic distribution of breast cancer-affected women in group 2 whose city of residence was known (n=657), color-coded by the recruiting centre.**


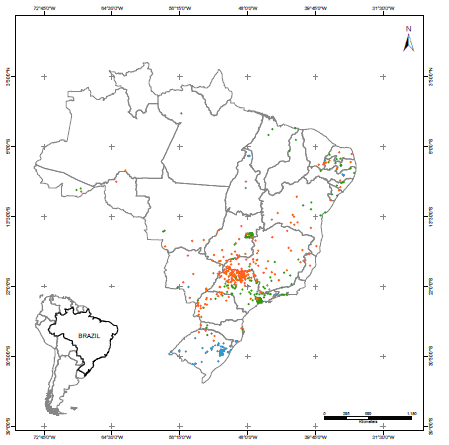


Legend: Blue, green and orange dots represent women from recruiting centres 1, 2 and 3, respectively.

**Figure S2. The detection of p.R337H by PCR-RFLP (*Restriction length fragment polymorphism*) and sequencing in homozygous and heterozygous p.R337H BC-affected women.**


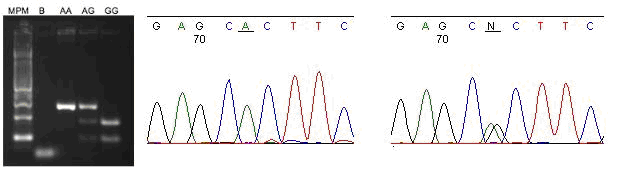


Legend: The DNA was amplified by PCR to generate a 238-base pair product encompassing exon 10 and the flanking splice sites. (a) RFLP analysis with *HhaI* (MPM = molecular weight marker; B = blank, the arrow indicates the uncleaved 238-base pair PCR product corresponding to the A allele). (b) Sequencing of a homozygous p.R337H carrier and (c) sequencing of a heterozygous p.R337H carrier (the underlined base corresponds to nucleotide 16,901 of the *TP53* gene).

**Table S1. *BRCA1, BRCA2 and CHEK2 1100delC* mutation status of the patients analyzed for these genes in the group 1.**

| **Patient** | **Clinical Criteria** | **BRCA1/2 (Seq)** | **BRCA1/2 (MLPA)** | **CHEK2 1100delC** |
| --- | --- | --- | --- | --- |
| 1* | HBOC | Negative | negative | NT |
| 2* | HBOC and HBCC | Negative | NT | negative |
| 3 | HBOC and HBCC | NT | negative | negative |
| 4 | HBCC | NT | positive | NT |
| 5 | HBOC | Negative | negative | NT |
| 6 | HBOC | NT | negative | NT |
| 7 | HBOC | Negative | negative | NT |
| 8 | HBOC | NT | negative | NT |
| 9 | HBOC | NT | positive | NT |
| 10 | HBOC | NT | negative | NT |
| 11 | HBOC | NT | negative | NT |
| 12 | HBOC | Positive | NT | NT |
| 13 | HBOC | Negative | NT | NT |
| 14 | HBOC | NT | positive | NT |
| 15 | HBOC and HBCC | NT | negative | negative |
| 16 | HBOC | NT | negative | NT |
| 17 | HBOC and HBCC | NT | NT | negative |
| 18 | HBOC and HBCC | NT | NT | negative |
| 19 | HBOC and HBCC | NT | NT | negative |

Legend: NT: not tested; **TP53* p.R337H mutation carriers. Molecular results of 30 cases of this group are not available.
